# Antibodies to Nipah-Like Virus in Bats (*Pteropus lylei)*, Cambodia

**DOI:** 10.3201/eid0809.010515

**Published:** 2002-09

**Authors:** James G. Olson, Charles Rupprecht, Pierre E. Rollin, Ung Sam An, Michael Niezgoda, Travis Clemins, Joe Walston, Thomas G. Ksiazek

**Affiliations:** * United States Naval Medical Research Unit Number 2, Phnom Penh, Cambodia; † Centers for Disease Control and Prevention, Atlanta, Georgia, USA; ‡ National Institute of Public Health, Ministry of Health, Phnom Penh, Cambodia; § Wildlife Conservation Society, Phnom Penh, Cambodia

**Keywords:** *Pteropus*, bats, antibodies, Nipah virus, Cambodia

## Abstract

Serum specimens from fruit bats were obtained at restaurants in Cambodia. We detected antibodies cross-reactive to Nipah virus by enzyme immunoassay in 11 (11.5%) of 96 Lyle’s flying foxes *(Pteropus lylei)*. Our study suggests that viruses closely related to Nipah or Hendra viruses are more widespread in Southeast Asia than previously documented.

 A large outbreak of encephalitis among swine farmers in Malaysia occurred from October 1998 to April 1999. Initially, *Japanese encephalitis virus* (JEV), a mosquito-borne pathogen endemic to the region, was suspected as the causative agent. However, a new paramyxovirus, Nipah virus, which is closely related to Hendra virus (HeV), was later implicated as the cause. Unlike JEV, Nipah virus predominated in adults rather than children. Nipah virus cases clustered in members of the same household, suggesting a high attack rate; in contrast, JEV causes symptomatic encephalitis in approximately 1/300 infected persons. A high proportion of Nipah virus patients had direct contact with pigs, unlike others in the same neighborhood who did not have the virus (providing evidence against a mosquito-borne disease); in addition, many of the pigs belonging to affected farmers had an associated history of illness (1–5).

 Clinically and epidemiologically, the Nipah virus cases in humans also differed from the few reported HeV infections (6). HeV is transmitted from horses, and two of three patients with HeV infections had severe respiratory involvement; only one patient had severe meningoencephalitis. In contrast, Nipah virus infections involved direct contact with pigs and predominant central nervous system disease, with only mild or undiagnosed clinical or radiologic evidence of pulmonary involvement. Incubation periods were <1 month. The main symptom was fever with headache, followed by rapid deterioration in consciousness (1,4).

 Nipah virus infection in pigs was frequently asymptomatic or, alternatively, occurred as an acute febrile illness with temperatures >40°C, accompanied by signs of respiratory and neurologic disease. Respiratory signs included open-mouth breathing, increased or forced respiration, and a harsh, nonproductive cough. Neurologic signs included head pressing, agitation and biting at bars, tetanic spasms, trembling, and muscle fasciculations (7,8).

 Comprehensive studies of domestic animals and wildlife showed that a substantial proportion of Malaysian fruit bats (genus *Pteropus*) had neutralizing antibodies to Nipah virus (7,9). Nipah virus was recently isolated from urine of Malaysian small flying foxes (*Pt. hypomelanus*) (10). HeV was detected in the four *Pteropus* spp. that occur in Australia, with a moderate (20%–25%) prevalence of HeV-neutralizing antibody (11). In addition, HeV was isolated from the grey-headed flying fox (*Pt. poliocephalus*) and black flying fox (*Pt. lecto*) (12). In preliminary studies in Indonesia, antibodies to Nipah-like viruses have been detected in other *Pteropus* spp. (T. Ksiazek, pers. comm.).

## The Study

 To further investigate the distribution of this new group of viruses, we investigated the prevalence of virus antibodies in other members of the genus *Pteropus* in Cambodia. In restaurants where bats are eaten in Phnom Penh, we collected 2-mL blood specimens from each bat as it was prepared for food. The restaurant owners purchased bats from a hunter who trapped them in Kampong Cham Province and transported them alive to restaurants in Phnom Penh. We stored the whole blood on wet ice for as long as 48 h, then transported it to the U.S. Naval Medical Research Unit No. 2, National Institute of Public Health Laboratory, in Phnom Penh on wet ice, and centrifuged it to separate the serum from the clot. Serum specimens were pipetted into screw-capped plastic vials and frozen at -20°C. Frozen serum specimens were sent from Cambodia to the Special Pathogens Branch, Division of Viral and Rickettsial Diseases, Centers for Disease Control and Prevention, Atlanta, Georgia. On arrival, the serum specimens were tested for antibodies to Nipah virus by enzyme immunoassay (EIA).

 Of 96 serum specimens from the fruit bat (*Pt. lylei*), 11 (11.5%) were positive (>1/10) for Nipah virus antibodies by EIA. All 11 were confirmed by serum neutralization test. Nine additional sera were found positive (low titers) only by neutralization assay. We also screened sera (when sufficient quantities were available) by neutralization test against HeV. In general, results were equivalent between the two tests. No sera were found positive for HeV and negative for Nipah virus. Our results suggest that the virus circulating in Cambodia is neither Nipah nor HeV, but another closely related virus.

## Conclusions

 Several species of the genus *Pteropus* show serologic evidence of Nipah or HeV infection. Attempts by several groups to recover virus from tissues of serologically positive bats have been unsuccessful, as have immunohistochemical tests to detect the infection in tissues (9). Several possible reasons may account for the inability to recover virus from serologically positive bats. Antibody-positive bats may represent the portion of those infected that survived and cleared the virus. Experimental inoculation of a small number of Australian *Pteropus* bats with a related paramyxovirus resulted in findings that the virus replicates, causes microscopic lesions, and is shed; the virus appears to clear as the antibody response appears (13,14). We did not attempt to isolate virus from blood, and our attempts to detect virus antigen in tissues by immunohistochemical tests in one bat were unsuccessful.

 We observed no evidence that HeV (15) or Nipah viruses move directly from bats to humans. However, during the outbreak of Nipah virus encephalitis outbreak in Malaysia, several laboratory-confirmed Nipah cases that lacked exposure to infected pigs were identified (P. Kitsutani, pers. comm.). In Cambodia, the distribution of *Pt. lylei* is limited to sites where they are protected from hunting, including urban areas and temples, where the human-bat interaction may be increased. The fact that these large bats are caught and used for food further increases the risk for exposure and infection in humans.

 Future studies should include an evaluation of the risk of Nipah virus infection among populations intensely exposed to bats, such as those who capture, transport, slaughter, and butcher bats, as well as bat rehabilitators, animal caretakers, and wildlife conservationists. We suggest that future studies also include a cross-sectional survey of swine in Cambodia. Unlike workers on the large, commercial swine production farms of Malaysia, typical swine farmers in Cambodia raise several swine for their own use and for the local market. The potential for amplification of the virus, unlike that observed in the large concentrated pig population in Malaysia, remains very limited. Finally, a systematic study of encephalitis causes may show whether Nipah virus causes disease in humans in Cambodia and elsewhere in the region.
